# Feco-prevalence, endoscopic pattern and associated factors of *Helicobacter Pylori* infection among symptomatic adult patients in Northern Tanzania

**DOI:** 10.1371/journal.pone.0307705

**Published:** 2024-07-22

**Authors:** Ibrahim Ali Ibrahim Muhina, Abid M. Sadiq, Fuad H. Said, Faryal M. Raza, Sarah K. Gharib, Sophia S. Muhali, Andrea R. Costantine, Mulhati S. Abdalla, Laura J. Shirima, Nyasatu G. Chamba, Furaha S. Lyamuya, Elifuraha W. Mkwizu, Kajiru G. Kilonzo, Venance P. Maro, Elichilia R. Shao

**Affiliations:** 1 Department of Internal Medicine, Kilimanjaro Christian Medical University College, Moshi, Tanzania; 2 Department of Internal Medicine, Kilimanjaro Christian Medical Centre (KCMC), Moshi, Tanzania; 3 Mnazi Mmoja Referral Hospital, Zanzibar, Tanzania; 4 Institute of Public Health, Kilimanjaro Christian Medical University College, Moshi, Tanzania; Texas Tech University Health Sciences Center, UNITED STATES OF AMERICA

## Abstract

**Background:**

Africa has consistently had the highest prevalence (70.1%) of *H*. *pylori*, and this has led to significant cases of dyspepsia, gastric cancers, and upper gastrointestinal bleeding. However, most studies have used sero-prevalence, which might not give the current state of the infection. Among the tests, the stool antigen test is simple, quick, and effective. The study aimed to determine the feco-prevalence, endoscopic pattern, and associated factors of *H*. *pylori* infection among symptomatic adult patients in Northern Tanzania.

**Materials and methods:**

A hospital-based, cross-sectional study was conducted from October 2022 to April 2023 among adults attending the gastroenterology clinic at Kilimanjaro Chistian Medical Centre. A systematic random sampling was used to select the participants with indications of undergoing esophagogastroduodenoscopy. Questionnaires, stool and blood samples, and endoscopy were used to collect variable data. Numerical and categorical variables were summarized into narrations and tables. Logistic regression was used to assess the factors associated with *H*. *pylori*.

**Results:**

The feco-prevalence of *H*. *pylori* was 43.4%. Chronic gastritis (51.1%) was the most common endoscopic pattern, whereas duodenal ulcers and gastric ulcers were significantly associated with *H*. *pylori* infection. Increasing in age (p <0.001) and blood group (p <0.001) were significantly associated with *H*. *pylori* infection in the adjusted analysis.

**Conclusion:**

The feco-prevalence of *H*. *pylori* is high in this setting. *H*. *pylori* stool antigen can be used as the initial workup for symptomatic patients before the initiation of proton pump inhibitors. Additionally, due to other causes of dyspepsia, it is advised that *H*. *pylori* stool antigen testing be part of the initial evaluation and esophagogastroduodenoscopy be considered in the absence of other alarm symptoms if symptoms persist despite an appropriate trial of medical therapy.

## Introduction

The most common chronic bacterial infection in the world is *H*. *pylori* [1, 2]. *H*. *pylori* infection is still a major public health concern around the world [3, 4]. According to the global systematic evaluation, almost 4.4 billion people were projected to be *H*. *pylori* positive in 2015 [3]. Africa has consistently recorded the greatest rate of *H*. *pylori* infection between 1970 and 2016, with a prevalence of 70.1% in 2019, followed by South America and Western Asia with prevalences of 69.4% and 66.6%, respectively [5].

Age, sex, ethnicity, socioeconomic characteristics, and geographic location all influence the prevalence of *H*. *pylori* [6]. When comparing the geographical distribution of *H*. *pylori*, developing countries have a larger sero-prevalence (> 90%) than developed countries [7]. According to the survey, affluent countries have a rate of 15.5%, while poor countries have a rate of 93.6% [8]. Tanzania is among the sub-Saharan African countries battling poverty, with 49.1% of its population living below the international poverty line of US $1.90 per day. US $1 is equivalent to 2,655/- Tanzanian shillings, and $1 can buy 1 liter of fresh milk or ½ liter of packaged milk [9]. The infection is assumed to be acquired in childhood due to the occurrence of disease in the first years of life, which rises significantly and then remains constant [10]. The likelihood of contracting an *H*. *pylori* infection increases as one gets older [7]. Other factors include hygienic practices, genetic predisposition, and population lifestyle [4, 6]. The spread of *H*. *pylori* is linked to low levels of education, unsanitary conditions (such as using untreated water), intra-familial clustering, high crowding indices, and tainted food (such as eating uncleaned raw fruits and vegetables) [10].

Both invasive and non-invasive tests can be used to detect *H*. *pylori* infections [11]. The non-invasive testing includes serology, urea breath tests, and stool antigen tests, whereas the invasive procedures include endoscopic gastric biopsy and rapid urease test (RUT) [12]. Among these tests, the stool antigen test is said to be simple, quick, and effective at both identifying a current infection and confirming eradication after *H*. *pylori* treatment. The stool antigen test detects the antigen of the bacterium and not the antibodies against it, which can be used for both diagnosis of the infection and monitoring therapy effectiveness [13]. The fecal antigen test is sensitive, specific, and affordable. Its sensitivity and specificity are more than 90% [14]. Like with the urea breath tests, false negative results can arise depending on the gastric disease and atrophic changes or exogenous factors such as the use of bismuth, proton-pump inhibitors, and antibiotics that reduce the bacterial load [15].

Patients who have *H*. *pylori* in their stomachs will have IgG antibodies in their serum, and these antibodies will continue to be present for a very long time after successful therapy. A study showed that negative conversion does not usually occur within one year and can take over three years, depending on the antibody titer [16]. Thus, it is not possible to distinguish between an active infection and an asymptomatic colonization state, or between a past and present *H*. *pylori* infection, using these serology-related parameters [17].

Polymerase Chain Reaction (PCR)-based detection of *H*. *pylori* can be invasive or non-invasive, depending on the sample acquired. This approach has great sensitivity and specificity (>95%). PCR detects *H*. *pylori* in a simple, accurate, rapid, automatic, and efficient manner, as it is more effective than other tests at detecting *H*. *pylori* in bleeding patients. However, PCR can produce false-positive results by detecting DNA fragments from bacteria that have been destroyed [18].

Notwithstanding these issues, research and data regarding the endoscopic patterns and feco-prevalence of *H*. *pylori* infections in symptomatic patients in Tanzania are scarce. Thus, among symptomatic individuals in northern Tanzania, the purpose of this study was to ascertain the feco-prevalence, endoscopic pattern, and associated factors of *H*. *pylori* infection.

## Materials and methods

### Study design and setting

This study was a hospital-based, analytical cross-sectional study conducted from 1^st^ October, 2022 to 30^th^ April, 2023. The study was conducted in the gastroenterology unit at KCMC in northern Tanzania. Permission to conduct the study was obtained from the Kilimanjaro Christian Medical University College Research and Ethics Committee with a research ethical clearance certificate number PG 168/2022 and from the head of the internal medicine department. Written consent was obtained from the participants after being informed of the purpose of the study. Confidentiality was observed, with adherence to the Helsinki Declaration, and all data was stored unlinked to patient identifiers. Each questionnaire had an ID number and not the name of the participant to ensure confidentiality. The information collected was used for study purposes only and was not shared with any unauthorized person. Participants who did not consent were not denied their right to medical care.

### Data collection and variables

A systematic random sampling was used to select the participants. A random starting point was selected, and patients were approached at a calculated interval of 2:1 at each clinical visit, whereby for every two patients selected, one was skipped and the technique repeated. The patients included presented with symptoms, particularly dyspepsia, as this refers to a heterogeneous group of symptoms that are localized in the epigastric region. Typical dyspeptic symptoms include postprandial fullness, early satiation, epigastric pain, and epigastric burning, but other upper gastrointestinal symptoms such as nausea, belching, abdominal bloating, or bleeding often occur [19].

The study included all adult patients (≥18 years) attending the gastroenterology unit at KCMC with indications of undergoing esophagogastroduodenoscopy (EGD), either as outpatients or inpatients in the hospital. EGD was performed in dyspeptic patients with alarming features such as age > 50 years, family history of upper gastrointestinal malignancy in a first-degree relative, unintended weight loss, gastrointestinal bleeding or iron deficiency anemia, dysphagia, odynophagia, persistent vomiting, and abnormal imaging suggesting organic disease [20].

This study excluded patients who were oxygen dependent, with anemia with Hb levels <4 g/dL, who had not fasted for the past eight hours, with low blood pressures of <90/60 mmHg, with tachycardia of >120 beats/min, and with a blood glucose level of <3.5 mmol/L or >14 mmol/L. Those exclusions were as per the guidelines of the pre-procedural checklist; hence, those who did not meet the pre-procedural checklist were excluded from this study. After an EGD, patients who were diagnosed with esophageal and/or gastric varices were excluded.

The dependent variable was *H*. *pylori* infection. Independent variables included age, sex, education level, residence, insurance status, marital status, occupation, living arrangement, hand hygiene, eating uncleaned raw fruits and vegetables, eating meat and/or fish, drinking tap water, hot tea consumption, coffee consumption, alcohol drinking, ABO blood group, body mass index, and endoscopic findings.

Before data collection, written informed consent was obtained from study participants. A questionnaire, weighing scale, stadiometer, stool container, blood sample for the blood group, and endoscopy were used to collect relevant data. Among eligible patients who consented, a face-to-face interview was administered, and information collected into the questionnaire included demographic characteristics, socio-economic status, anthropometric values, personal habits, and blood group. The questionnaire was developed in Swahili (the local language). Then, the responses were translated back into English. Data quality assurance and control were done at the end of every day by the principle investigator to observe the completeness, accuracy, relevance, and timeliness of the data based on the intended variables to be collected.

The blood and stool samples were collected from the participants and sent to the laboratory immediately for processing. A special, clean stool container was used to collect the stool sample from each participant separately. *H*. *pylori* Antigen Rapid Test Cassette (Feces) was used to determine *H*. *pylori* status with a relative sensitivity of >99.9% and a relative specificity of >99.9%. Cross reactivity has been studied at 1x 10^9^ organisms/ml, as the Antigen Rapid Test Cassette (Feces) has been designed to detect *H*. *pylori* through visual interpretation of color development in the internal strip. The membrane was immobilized with an anti-*H*. *pylori* monoclonal antibody on the test region. During the test, the specimen was allowed to react with an anti-*H*. *pylori* monoclonal antibody conjugated with colored particles, which were precoated on the sample pad of the test. If there are enough *H*. *pylori* antigens in specimens, a colored band will form at the test region of the membrane. The presence of this colored band indicates a positive result, while its absence indicates a negative result. In addition, for participants who self-reported their blood group with a recorded verification, their ABO system was laboratory determined. The ABO and Rh blood groups were determined by a slide agglutination test using monoclonal anti-A, anti-B, anti-AB, and anti-D (Rh) antibodies according to the manufacturer’s recommendation.

### Statistical analysis

STATA version 17 was used for data cleaning and analysis. Numerical variables were summarized into mean/median and standard deviation/interquartile range. Categorical variables were summarized into frequency and percentage. A cross-tabulation of *H*. *pylori* infection by endoscopic findings together with a chi-square test was used to determine the endoscopic pattern and its association with *H*. *pylori* infection. A crude logistic regression was used to identify factors associated with *H*. *pylori* infection, whereby variables with a p-value of <0.05 were considered statistically significant, together with their 95% CI. Variables identified as potential confounders were considered for multivariable logistic regression to obtain the adjusted odd ratio.

## Results

A total of 389 patients were selected. Among these, nine patients did not consent to an EGD; hence, 380 patients were analyzed with a response rate of 97.7%. Among the 380 participants in the study, 167 (44.4%) were over the age of 50, with a median age of 47 (IQR 31–63) years. More than half (55.8%) were women, 250 (66.5%) were married or cohabiting, and 143 (37.6%) had a primary education. Patients who earned less than TZS 100,000 per month were 172 (46.2%), and 147 (38.9%) were unemployed. The majority of study participants (94.5%) reported tapped water as their source of drinking water, while 215 (56.6%) reported not treating their drinking water (by chemical methods or boiling or filtering) (Table 1).

**Table 1:** Social-demographic characteristics of study participants (n = 380).

Variable	n	%
Age (years); *median [IQR] 47 [31–63]*		
18–30	92	24.5
31–40	61	16.2
41–50	56	14.9
>50	167	44.4
Sex		
Male	168	44.2
Female	212	55.8
Education		
No formal education	12	3.2
Primary education	143	37.6
Secondary education	103	27.1
High level of education	122	32.1
Marital status		
Single/divorced	103	27.4
Married/cohabiting	250	66.5
Widow/widower	23	6.1
Employment status		
Unemployed	147	38.9
Employed	93	24.6
Self-employed	138	36.5
Income (TZS)		
<100,000	172	46.2
100,000–500,000	143	38.4
>500,000	57	15.3
Area of residence		
Rural	216	43.2
Urban	164	56.8
Household size; *median [IQR] 5 [3–6]*		
≤3	105	28.3
4–5	157	42.3
≥6	109	29.4
People per room		
1	62	16.4
2–3	297	78.4
≥4	20	5.3
Source of drinking water		
Tapped water	358	94.5
Other sources	21	5.5
Treat drinking water		
Yes	165	43.4
** Chemical**	94	57.0
** Boiling/filtering**	71	43.0
No	215	56.6

TZS: Tanzanian shillings

The proportion of *H*. *pylori* stool antigen tests that were positive was 43.4%. Out of 380 study participants, 193 (51.0%) had chronic gastritis, 115 (30.3%) had gastro-esophageal reflux disease (GERD), 54 (14.2%) had duodenitis, 42 (11.5%) had acute gastritis, and 34 (9%) had a duodenal ulcer. There was a statistical significance in *H*. *pylori* infection between 29 (85.3%) patients with duodenal ulcers (p <0.001) and 18 (81.8%) patients with gastric ulcers (p <0.001) (Table 2).

**Table 2:** Association of *H*. *pylori* infection and endoscopic findings (n = 380).

Pattern	Total	H. Pylori		p value
	n (%)	Positive; n (%)	Negative; n (%)	
Acute gastritis	42 (11.5)	13 (31.0)	29 (69.0)	0.084
GERD	115 (30.3)	46 (40.0)	69 (60.5)	0.375
Duodenal ulcer	34 (9.0)	29 (85.3)	5 (14.7)	<0.001
Duodenal tumor	3 (0.8)	1 (33.3)	2 (66.7)	1.000
Gastric ulcer	22 (5.8)	18 (81.8)	4 (18.2)	<0.001
Gastric tumor	17 (4.5)	5 (29.4)	12 (70.6)	0.233
Esophageal tumor	7 (1.8)	2 (28.6)	5 (71.4)	0.704
Esophageal ulcer	5 (1.3)	3 (60.0)	2 (40.0)	0.656
Esophagitis	14 (3.7)	6 (42.9)	8 (57.1)	0.965
Chronic gastritis	194 (51.1)	90 (46.4)	104 (53.6)	0.233
Duodenitis	54 (14.2)	24 (44.4)	30 (55.6)	0.870
Candidiasis	26 (6.8)	14 (53.8)	12 (46.2)	0.266
Diaphragmatic hernia	17 (4.5)	6 (35.3)	11 (64.7)	0.489
Polyp of stomach	1 (0.3)	1 (100)	0 (0)	0.434
Polyp of duodenum	1 (0.3)	1 (100)	0 (0)	0.434

GERD = Gastro-esophageal reflux disease

In adjusted analysis, age and blood group were significantly associated with *H*. *pylori* infection. The odds of *H*. *pylori* infection increase by 1.98 times for every one-year increase in age (aOR: 1.98, 95% CI: 1.60–2.46, p <0.001). When compared to blood group O, only blood group A (aOR: 0.12, 95% CI: 0.04–0.38, p <0.001) had lower odds of *H*. *pylori* infection, while blood group B (aOR: 0.30, 95% CI: 0.09–1.00, p 0.050) and blood group AB (aOR: 0.35, 95% CI: 0.11–1.16, p 0.086) were not statistically significant (Table 3).

**Table 3:** Factors associated with positive stool antigen test for *H*. *pylori* (n = 380).

Variable	Stool antigen test		cOR (95% CI)	p value	aOR (95% CI)	p value
	Positive n (%)	Negative n (%)				
Age (years)			1.03 (1.01–1.04)	<0.001	1.98 (1.60–2.46)	<0.001
Sex						
** **Male	70 (41.7)	98 (58.3)	1			
** **Female	95 (44.8)	117 (55.2)	1.14 (0.75–1.71)	0.539	1.16 (0.72–1.88)	0.549
Education						
** **No formal education	4 (33.3)	8 (66.7)	1			
** **Primary education	66 (46.2)	77 (53.8)	1.54 (0.44–5.38)	0.502	1.43 (0.35–5.81)	0.616
** **Secondary education	39 (40.6)	57 (59.4)	0.90 (0.55–1.46)	0.658	0.65 (0.33–1.27)	0.209
** **Higher level education	53 (43.4)	69 (56.6)	1.12 (0.65–1.93)	0.676	0.92 (0.48–1.75)	0.796
Marital status						
** **Single/divorced	47 (45.6)	56 (54.4)	1		1	
** **Married/cohabiting	107 (42.8)	143 (57.2)	0.92 (0.37–2.28)	0.851	0.61 (0.22–1.73)	0.352
** **Widow/widower	10 (43.5)	13 (56.5)	1.03 (0.44–2.43)	0.950	0.75 (0.29–1.94)	0.550
Income (TZS)						
** **<100000	75 (43.6)	97 (56.4)	1			
** **100000–500000	64 (44.8)	79 (55.2)	0.88 (0.48–1.61)	0.667	0.68 (0.30–1.52)	0.345
** **>500000	23 (40.4)	34 (59.7)	0.84 (0.49–1.56)	0.571	0.61 (0.30–1.26)	0.182
Area of residence						
** **Rural	74 (45.1)	90 (54.9)	1			
** **Urban	91 (42.1)	125 (57.9)	0.89 (0.59–1.33)	0.560	1.35 (0.84–2.16)	0.215
Household Size						
** **≤3	47 (44.8)	58 (55.2)	1			
** **4–5	72 (45.9)	85 (54.1)	0.77 (0.45–1.33)	0.356	0.87 (0.46–1.63)	0.668
** **≥6	42 (38.5)	67 (61.5)	0.74 (0.45–1.22)	0.235	0.65 (0.37–1.16)	0.143
Source of drinking water						
** **Tapped water	155 (43.3)	203 (56.7)	1			
** **Other sources	10 (47.6)	11 (52.4)	1.19 (0.49–2.88)	0.698	0.76 (0.27–2.11)	0.592
Treat drinking water						
** **Yes	72 (43.6)	93 (56.4)	1			
** **No	93 (43.3)	122 (56.7)	0.98 (0.65–1.48)	0.941	1.03 (0.62–1.69)	0.914
Eat uncleaned raw vegetable						
** **No	13 (52.0)	12 (48.0)	1			
** **Yes	152 (42.8)	203 (57.2)	0.69 (0.31–1.55)	0.373	0.74 (0.29–1.90)	0.536
BMI						
** **Underweight	13 (44.8)	16 (55.2)	1			
** **Normal weight	75 (44.6)	93 (55.4)	1.42 (0.55–3.65)	0.472	1.44 (0.48–4.36)	0.516
** **Overweight	54 (38.6)	86 (61.4)	1.43 (0.73–2.79)	0.301	1.41 (0.65–3.05)	0.381
** **Obese	23 (53.5)	20 (46.5)	1.83 (0.92–3.65)	0.085	1.80 (0.82–3.93)	0.143
Blood group						
** **O	99 (56.6)	76 (43.4)	1		1	
** **A	30 (36.6)	52 (63.4)	0.11 (0.04–0.31)	<0.001	0.12 (0.04–0.38)	<0.001
** **B	30 (35.3)	55 (64.7)	0.24 (0.08–0.75)	0.014	0.30 (0.09–1.00)	0.050
** **AB	4 (12.1)	29 (87.9)	0.25 (0.08–0.79)	0.018	0.35 (0.11–1.16)	0.086

BMI: Body mass index; TZS: Tanzanian shillings

## Discussion

The feco-prevalence of *H*. *pylori* in this study was 43.4%, and the common endoscopic patterns were acute and chronic gastritis, GERD, and duodenitis. Chronic gastritis turned out to be the most common endoscopic pattern, while gastric and duodenal ulcers showed a significant correlation with *H*. *pylori* infection. Increasing age and blood group O are substantially correlated with *H*. *pylori* infection.

This study’s prevalence (43.4%) was higher than that of developed countries, including the United States of America (35.6%), Switzerland (18.9%), Sweden (26.2%), Norway (30.7%), and New Zealand (24.0%) [3]. In comparison to reports from other developing nations, such as Nigeria (87.7%), South Africa (77.6%), the Democratic Republic of Congo (77.4%), and Tunisia (72.8%) [3], the prevalence of this study was low. The level of urbanization, sanitation, access to clean water, and varying economic positions are likely contributing factors to these variations in *H*. *pylori* prevalence. Even within Tanzania, the prevalence of *H*. *pylori* varies significantly between regions [3].

The previous study conducted in Tanzania ten years ago reported a prevalence of 65.0% using RUT through gastric antral biopsies. It is possible that the current prevalence has dropped as a result of readily available diagnostic tools that have allowed for early treatment and eradication, access to clean water, improved socio-economic status, and better sanitation [21]. A study in Kenya had higher results, reporting that 54.8% of adults had *H*. *pylori* antigen-positive results [22]. A tertiary hospital in western Tanzania reported that the sero-prevalence of *H*. *pylori* was 39.1% [7]. Due to the high prevalence of *H*. *pylori* infection in developing countries like Tanzania, the extensive use of proton-pump inhibitors prior to *H*. *pylori* testing and EGD may lead to improper diagnosis and, hence, ineffective treatment among dyspeptic patients. This is a concern because the World Gastroenterology Organization advises a test-and-treat strategy for dyspeptic patients in areas with a high frequency of the condition in order to stop the development of gastric cancer [12]. This is crucial, especially in low-resource countries like Tanzania, where there aren’t enough resources to deal with the burden of cancer.

The majority of symptomatic patients in our study had organic reasons for dyspepsia that were identified through endoscopy. Of the 380 participants in the study, 42 (11.5%) had acute gastritis, 115 (30.3%) had GERD, 34 (9%) had a duodenal ulcer, and 194 (51.1%) had chronic gastritis. *H*. *pylori* infection was significantly associated with gastric and duodenal ulcers. *H*. *pylori* infection was found in 85.3% of patients with duodenal ulcers and 81.8% of patients with gastric ulcers. In our study, endoscopic evidence of chronic gastritis was present in slightly more than half of the symptomatic patients. However, there was no statistically significant link between *H*. *pylori* infection and gastritis. These findings contradict literature-based claims that the bacteria is a substantial contributor to gastritis. Similar to the findings of a study in Kenya, gastritis was discovered to be the most prevalent endoscopic abnormality in that study population [23]. Our study also showed that there is a statistically significant correlation between *H*. *pylori* infection and gastric and duodenal ulcers.

Knowing the common causes of dyspepsia and how frequently *H*. *pylori* infections occur is crucial for clinicians working in settings with few resources, particularly when access to endoscopic services is restricted. Dyspepsia symptoms do not consistently identify people with malignancy or other significant upper gastrointestinal diseases. Therefore, patients with dyspepsia might have multiple etiologies that could be detected with endoscopy or other procedures that have been classified using patient age and other alert signs. Due to the multiple etiologies, dyspepsia presentations need thorough workups and specialized care to avoid missing a disease condition that is treatable at an early stage.

In this study, increased age was correlated with *H*. *pylori* feco-prevalence (p <0.001). There are conflicting findings about the relationship between the aforementioned risk factors and *H*. *pylori* infection. One of the key elements in the development of *H*. *pylori* is age. A substantial correlation between this factor and the prevalence of *H*. *pylori* infection has been found in numerous recent studies. In line with a study conducted in western Tanzania, the probabilities of having *H*. *pylori* infection increased with age in this study. Similar results were obtained in China, which used the Carbon-13 Urease Breath Test to assess the prevalence of *H*. *pylori* and found that the infection increased with age, peaking in the age group of 51–60 years [24].

This could be explained by the fact that increasing in age causes a number of changes in biological factors, including the loss of telomeres, the activity and metabolism of stem cells, the increase in biological and environmental stress, the malfunctioning of many micro- and macro-molecules and the cell cycle, and a weakened immune system [25]. In contrast, a study conducted in Ethiopia found no significant relationship between age and the prevalence of *H*. *pylori* [4].

In our study, different blood group systems exhibited reduced risks of *H*. *pylori* infection compared to blood group O. Similar findings in Pakistan found a substantial relationship between *H*. *pylori* infection and blood group O [26]. The results of a meta-analysis of 30 studies on the correlation between the O blood group and *H*. *pylori* infection showed that those with the O blood group had higher probabilities of developing the infection than people with the non-O blood group [27]. Blood group antigens are known to increase the chance of acquiring gastric cancer and peptic ulcer disease [28]. For a long time, blood group O had been linked to duodenal ulcers and blood group A had been linked to gastric cancer, but no reason for these associations could be established [2]. Recent research has shown that the Lewisb antigen, which is primarily prevalent in blood type O, serves as a receptor for *H*. *pylori* adhesins and mediates bacterial adherence to the gastric surface necessary for bacterial colonization [27]. Blood group O individuals may be more likely to develop peptic ulcer disease than those of other blood groups, possibly as a result of a larger density of colonized *H*. *pylori*. Additionally, the Lewisb antigen cannot attach to *H*. *pylori* when blood types A, B, and AB are substituted. Lower *H*. *pylori* infection rates in people with blood group O may be the result of decreased Lewisb antigen exposure [28].

The study limitations were that this study and its findings were in a selected symptomatic population. Therefore, the feco-prevalence among symptomatic patients in this study might be overestimated. The study did not include adult dyspeptic patients visiting other health centers in the region. Patients were not assessed for the intake of non-steroidal anti-inflammatory drugs, as some studies have suggested that they can affect the susceptibility to *H*. *pylori*. The study was hospital-based, so the findings cannot be generalized to general populations. The study design was cross-sectional; hence, we cannot establish the causal effect between dyspepsia and positive feco-prevalence among study participants. Moreover, this study was limited only to symptomatic patients visiting the hospital. Therefore, it might not show the actual prevalence of the infection in the region.

## Conclusion

In conclusion, the feco-prevalence of *H*. *pylori* infection in this study was higher compared to developed countries but lower compared to developing countries. Chronic gastritis was the commonest endoscopic pattern but was not associated with fecal *H*. *pylori* positivity. *H*. *pylori* feco-prevalence was significantly associated with gastric and duodenal ulcers endoscopically. Increasing in age and blood group O were the risk factors with a significant association with fecal *H*. *pylori* positivity. The present findings have potential implications for clinical practice and public health in the study area. It is recommended that all dyspeptic patients be tested for *H*. *pylori* stool antigen prior to proton-pump inhibitors and/or antibiotic use, depending on the stool antigen results. If symptoms persist, further workup through an EGD will help in the early diagnosis and appropriately treatment outcomes, hence unnecessary antibiotic use, and reduce the risks of antimicrobial resistance. EGD will also help with the early diagnosis of other gastrointestinal conditions, such as gastric tumors, and early treatment before metastasis.
